# Common variants of pro-inflammatory gene *IL1B* and interactions with *PPP1R13L* and *POLR1G* in relation to lung cancer among Northeast Chinese

**DOI:** 10.1038/s41598-023-34069-z

**Published:** 2023-05-05

**Authors:** Jiaoyang Yin, Chunhong Wang, Ulla Vogel, Yegang Ma, Ying Zhang, Huiwen Wang, Zhenxiang Sun, Shuai Du

**Affiliations:** 1 Key Laboratory of Environment and Population Health of Liaoning Education Ministry (Shenyang Medical College), Shenyang, 110034 People’s Republic of China; 2 Basic Medical School, Shenyang Medical College, Shenyang, 110034 People’s Republic of China; 3 National Research Centre for the Working Environment, 2100 Copenhagen, Denmark; 4 Department of Thoracic Surgery, Liaoning Cancer Hospital, Shenyang, 110042 People’s Republic of China; 5 College of Information, Liaoning University, Shenyang, 110036 People’s Republic of China

**Keywords:** Cancer, Biomarkers, Molecular medicine

## Abstract

Lung cancer is a complex disease influenced by a variety of genetic and environmental factors. The cytokine interleukin 1 encoded by *IL1B* is an important mediator of the inflammatory response, and is involved in a variety of cellular activities. The effect of single nucleotide polymorphisms (SNP) at *IL1B* has been investigated in relation to cancer with inconsistent results. This Northeastern-Chinese case–control study involving 627 cases and 633 controls evaluated the role of three haplotype-tagging single nucleotide polymorphisms (htSNP) (rs1143633, rs3136558 and rs1143630) representing 95% of the common haplotype diversity across the *IL1B* gene and assessed interactions with *IL1B*, *PPP1R13L*, *POLR1G* and smoking duration in relation to lung cancer risk. The analyses of five genetic models showed associations with lung cancer risk for rs1143633 in the dominant model [adjusted-OR (95% CI) = 0.67 (0.52–0.85), *P* = 0.0012] and rs3136558 in the recessive model [adjusted-OR (95% CI) = 1.44 (1.05–1.98), *P* = 0.025]. Haplotype4 was associated with increased lung cancer risk [adjusted-OR (95% CI) = 1.55 (1.07–2.24), *P* = 0.021]. The variant G-allele of rs1143633 was protective in smoking sub-group of > 20 years. Using multifactor dimensionality reduction (MDR) analyses, we identified the three best candidate models of interactions and smoking-duration or *IL1B* rs1143633 as main effect. In conclusion, our findings suggest that *IL1B* SNP rs1143633 may associate with lower risk of lung cancer, confirming previously identified marker; *IL1B* SNP rs3136558 and haplotype4 consisting of *IL1B* htSNPs may associate with increasing risk of lung cancer; interactions of *IL1B* with *POLR1G* or *PPP1R13L* or smoking-duration, which is independent or combined, may involve in risk of lung cancer and lung squamous cell carcinoma.

## Introduction

Lung cancer is an important and prevalent cause of cancer-related death worldwide and constitutes a serious public health problem. Lung cancer is a complex disease influenced by a variety of genetic and environmental factors. Susceptibility gene/single nucleotide polymorphisms (SNP) have been linked to lung cancer risks. Tobacco remains the leading risk factor for lung cancer^1^. Chronic lung diseases that entail chronic inflammation have been suspected to play a role in the pathogenesis of lung cancer^1^. Another possible mechanism may involve gene–gene or gene–environment interactions in relation to lung cancer^2^.

*IL1B* (interleukin 1 beta) (HGNC ID: 5992, Gene ID: 3553) is located on chromosome 2q14.1. The protein encoded by *IL1B* is a member of the interleukin 1 cytokine family (https://www.ncbi.nlm.nih.gov/gene/3553). The *IL1* cluster consists of three related genes: *IL1A*, *IL1B*, and *IL1RA*, which encode the signal proteins IL1A, IL1B, and their receptor, IL1RA, respectively^3^. IL1A and IL1B are pro-inflammatory cytokines, whereas IL1RA is an anti-inflammatory cytokine and competes with IL1A and IL1B for binding to the IL1 receptors^4^. The interleukin 1 cytokine is an important mediator of the inflammatory response, and is involved in a variety of cellular activities, including cell proliferation, differentiation, apoptosis (https://www.ncbi.nlm.nih.gov/gene/3553) and innate immunity^5^. Cytokines are known as important regulators in cancer and are involved in inflammatory and immunological responses^6^.

The *IL1B* gene has been extensively investigated in relation to cancers and inflammatory and infectious diseases. The effect of SNPs at *IL1B* has been investigated in relation to cancer with inconsistent results in predominantly European and Asian populations^6–13^. Polymorphisms in cytokine genes may be functional^14^, modify the inflammatory and immune responses and therefore modulate risk of lung cancer. Non-functional polymorphisms may be genetically linked to functional polymorphisms. There are only few studies involving *IL1B* SNPs and lung cancer risk, retrieving only one study of Caucasia-Danes^7^. The molecular mechanism underlying carcinogenic IL1B inflammatory-induced lung cancer is still unclear completely.

Two genes *PPP1R13L* [protein phosphatase 1 regulatory subunit 13 like] (HGNC ID: 18838, Gene ID: 10848) and *POLR1G* [RNA polymerase I subunit G, Previous symbols CD3EAP: CD3e molecule, epsilon-associated protein] (HGNC ID: 24219, Gene ID: 10849) located on chromosome 19q13.32 relate to DNA repair and cell survival and cell proliferation, respectively. We previously reported that *PPP1R13L* rs1970764, *POLR1G* rs967591 and rs735482 were associated with lung cancer or interacted in relation to lung cancer risk among both Caucasian Danes and Chinese^15–18^. *IL1B*, *PPP1R13L* and *POLR1G* had the effect on apoptosis and DNA repair pathways.

*IL1B* belongs to pathways of cytokines and inflammatory response, immune system, and lung fibrosis. *PPP1R13L* and *POLR1G* share pathway of gene expression (transcription) [https://www.ncbi.nlm.nih.gov/gene/3553, /10848, /10849]. Significant advances in statistical approaches make it possible considering the main pathways to which the genes belongs and possible covariates, as required in the analysis of complex traits^19^. A deeper understanding of common pathways of inflammation and cancer may increase our understanding of the role of inflammation and cancer^20^.

In this case–control study of Northeastern-Chinese, we explored the role of haplotype-tagging single nucleotide polymorphisms (htSNP) tagging 95% of common haplotypes across the *IL1B* gene on lung cancer risk. And we assessed gene–gene or gene–gene–environment interactions between genes of pathway of cytokines and inflammatory response and gene expression (transcription) pathway related to lung cancer risk, including the interaction between *IL1B* htSNPs, *PPP1R13L* and *POLR1G* risk SNPs and smoking-duration.

## Results

### Population characteristics

The baseline characteristics of the lung cancer patients and healthy controls are shown in Table 1. The *IL1B* three htSNPs were evaluated in a Northeastern-Chinese hospital-based case–control study involving 627 lung cancer cases and 633 controls. There were no statistically significant differences for the distribution of age and sex between cases and controls. However, there were more cases with family history of cancer and longer smoking history (> 20 years) (both *P* < 0.0001).

**Table 1:** Distribution of selected characteristics in Chinese case–control study population.

Characteristics	Cases n (%)	Controls n (%)	*P* value
Over all	627	633	
Age (years)			
Mean (± SD)	58 (± 10.4)	58 (± 10.5)	0.846^a^
≤ 40	29 (4.6%)	29 (4.6%)	
41–50	109 (17.4%)	125 (19.7%)	
51–60	222 (35.4%)	214 (33.8%)	0.748^b^
> 60	267 (42.6%)	265 (41.9%)	
Sex			
Female	185 (29.5%)	184 (29.1%)	
Male	442 (70.5%)	449 (70.9%)	0.86^b^
Family history of cancer			
No	536 (85.5%)	628 (99.2%)	
Yes	91 (14.5%)	5 (0.8%)	**< 0.0001**^b^
Smoking duration			
Never	241 (38.4%)	333 (52.6%)	
≤ 20 (years)	104 (16.6%)	98 (15.5%)	**< 0.0001**^b^
> 20 (years)	282 (45.0%)	202 (31.9%)	
Histopathology			
Lung squamous cell carcinoma	266 (42.4%)		
Lung adenocarcinoma	251(40.0%)		
Other	110 (17.6%)		

^a^For Mann–Whitney U test (*P* < 0.0001 for Shapiro–Wilk test).

^b^For χ^2^ test (two-sided), boldface indicates statistical significance.

The minor-allele frequencies were determined among the controls (G = 0.46, C = 0.38 and A = 0.16 for rs1143633, rs3136558 and rs1143630, respectively). The minor-allele frequency of rs1143633 was similar to one in HapMap-HCB (Han Chinese in Beijing) reported by NCBI dbSNP database (https://www.ncbi.nlm.nih.gov/snp) (*P* = 0.745) whereas rs3136558 (*P* = 0.002) and rs1143630 (*P* = 0.027) were not (Table 2). The genotype distributions in the control group were in Hardy–Weinberg equilibrium for rs1143633 (*P* = 0.29), rs3136558 (*P* = 0.26), and rs1143630 (*P* = 0.77).

**Table 2:** Characteristics of *IL1B* three htSNPs selected and SNPs in *PPP1R13L* and *POLR1G*.

Gene/chromosome/rs^a^	Position	Functional consequence	Base change	Allele frequency in HapMap HCB^b^	Control MAF^c^ in this study
*IL1B* Chr2q14.1					
rs1143633	112832890	Intron	A/G	A0.5465/G0.4535	G0.46
rs3136558	112833698	Intron	T/C	T0.7442/C0.2558	C0.38
rs1143630	112834078	Intron	C/A	C0.9024/A0.0976	A0.16
*PPP1R13L* Chr19q13.32					
rs1970764	45387615	Intron	A/G	No	G0.48^d^
*POLR1G* Chr19q13.32					
rs967591	45406676	5′ UTR	G/A	G0.5250/A0.4750^e^	A0.42^d^
rs735482	45408744	Exon, missense	A/C	A0.5581/C0.4419	C0.45^d^

^a^Information from NCBI SNP database (GRCh38.p12) and HapMap database. From 3′–5′.

^b^Han Chinese in Beijing.

^c^Minor allele frequency.

^d^From previous result (Yin et al.^18^), here this is employed for interaction analysis.

^e^CHB + JPT (Han Chinese in Beijing + Japanese from 1000GENOMES).

### Selected *IL1B* htSNPs and lung cancer risk

Genotype distributions and lung cancer risk for the three *IL1B* htSNPs in co-dominant, dominant, recessive, over-dominant and log-additive models after adjustment smoking-duration were analyzed (Table 3). For whole study group, rs1143633 in the dominant model [Odd Ratio (95% confidence interval): adjusted-OR (95% CI) = 0.67 (0.52–0.85), *P* = 0.0012] (and also including co-dominant model and log-additive model) and rs3136558 in the recessive model [adjusted-OR (95% CI) = 1.44 (1.05–1.98), *P* = 0.025] (and also including co-dominant model) showed in association with lung cancer risk. No significant models with association were found for rs1143630. For subgroup stratified by smoking duration, G variant-allele of rs1143633 showed the protective effect in > 20 years subgroup (Table 4). No significant associations were found for two other htSNPs (data not shown). For subgroup stratified by histopathology, rs1143633 in the log-additive model [adjusted-OR (95% CI) = 0.59 (0.47–0.75), *P* < 0.0001] and rs3136558 in the log-additive model [adjusted-OR (95% CI) = 1.35 (1.08–1.69), *P* = 0.0086] were associated with the disease risk in the subgroup of lung squamous cell carcinoma (Table 3). No significant association was found for subgroups of lung adenocarcinoma and other histoathology (data not shown).

**Table 3:** Associations of single htSNP in *IL1B* with lung cancer risk.

Type/model^a^/rs	Genotype	Controls 633 n (%)	Cases 627 n (%)	OR (95 CI)^b^^,c^	*P* value^c^	AIC^d^
Whole group: lung cancer						
rs1143633 (A>G)						
Co-dominant	A/A	169 (28.2%)	224 (36.4%)	1.00		
	G/A	311 (51.9%)	289 (47%)	**0.68 (0.53–0.89)**	**0.0044**	1651.4
	G/G	119 (19.9%)	102 (16.6%)	**0.62 (0.44–0.87)**		
Dominant	A/A	169 (28.2%)	224 (36.4%)	1.00		
	G/A-G/G	430 (71.8%)	391 (63.6%)	**0.67 (0.52–0.85)**	**0.0012**	1649.7
Recessive	A/A-G/A	480 (80.1%)	513 (83.4%)	1.00		
	G/G	119 (19.9%)	102 (16.6%)	0.78 (0.58–1.05)	0.1	1657.6
Over-dominant	A/A-G/G	288 (48.1%)	326 (53%)	1.00		
	G/A	311 (51.9%)	289 (47%)	0.81 (0.65–1.02)	0.077	1657.1
Log-additive	–	–	–	**0.77 (0.65–0.91)**	**0.0021**	1650.8
rs3136558 (T>C)						
Co-dominant	T/T	223 (37.2%)	214 (34.8%)	1.00		
	C/T	296 (49.4%)	293 (47.6%)	1.02 (0.79–1.31)	0.079	1657.2
	C/C	80 (13.4%)	108 (17.6%)	**1.46 (1.03–2.06)**		
Dominant	T/T	223 (37.2%)	214 (34.8%)	1.00		
	C/T-C/C	376 (62.8%)	401 (65.2%)	1.11 (0.88–1.41)	0.38	1659.5
Recessive	T/T-C/T	519 (86.6%)	507 (82.4%)	1.00		
	C/C	80 (13.4%)	108 (17.6%)	**1.44 (1.05–1.98)**	**0.025**	1655.2
Over-dominant	T/T-C/C	303 (50.6%)	322 (52.4%)	1.00		
	C/T	296 (49.4%)	293 (47.6%)	0.91 (0.73–1.15)	0.44	1659.6
Log-additive	–	–	–	1.16 (0.99–1.37)	0.073	1657
rs1143630 (C>A)						
Co-dominant	C/C	422 (70%)	407 (66.9%)	1.00		
	A/C	164 (27.2%)	190 (31.2%)	1.19 (0.92–1.53)	0.21	1655.7
	A/A	17 (2.8%)	11 (1.8%)	0.67 (0.31–1.46)		
Dominant	C/C	422 (70%)	407 (66.9%)	1.00		
	A/C-A/A	181 (30%)	201 (33.1%)	1.14 (0.89–1.46)	0.29	1655.7
Recessive	C/C-A/C	586 (97.2%)	597 (98.2%)	1.00		
	A/A	17 (2.8%)	11 (1.8%)	0.64 (0.29–1.38)	0.25	1655.5
Over-dominant	C/C-A/A	439 (72.8%)	418 (68.8%)	1.00		
	A/C	164 (27.2%)	190 (31.2%)	1.21 (0.94–1.55)	0.15	1654.7
Log-additive	–	–	–	1.07 (0.86–1.33)	0.54	1656.5
Histopahtology subgroup: lung squamous cell carcinoma^e^						
rs1143633 (A>G)						
Log-additive	–	–	–	0.59 (0.47–0.75)	< 0.0001	980.8
rs3136558 (T>C)						
Log-additive	–	–	–	1.35 (1.08–1.69)	0.0086	994.5

^a^Dominant model: AB (Heterozygote) + BB (Homozygous variant-type) versus AA (Homozygous wild-type), Recessive model: BB versus AA + AB, Co-dominant model: AB versus AA and BB versus AA, Over-dominant model: AB versus AA + BB, Log-additive model: Analysis of trend where AA is ‘0’, AB is ‘1’ and BB is ‘2’.

^b^OR (odd ratio), 95% CI (95% confidence interval), adjusted for smoking duration.

^c^Boldface indicates statistical significance.

^d^Akaike’s Information Criterion.

^e^Only list the best significant models.

**Table 4:** rs1143633's genotypes within smoking-duration.

Smoking-duration	Genotype	Controls	Cases	OR (95% CI)	*P* value
Never	AA	96	87	1.0	
	GA	163	110	0.75 (0.51–1.09)	0.126
	GG	60	38	0.70 (0.42–1.15)	0.159
	GA + GG	223	148	0.73 (0.51–1.05)	0.087
≤ 20 (years)	AA	30	39	1.00	
	GA	44	49	0.86 (0.46–1.60)	0.628
	GG	17	13	0.59 (0.25–1.40)	0.227
	GA + GG	61	62	0.78 (0.43–1.41)	0.415
> 20 (years)	AA	43	98	1.00	
	GA	104	130	**0.55 (0.35–0.85)**^**a**^	**0.007**^**a**^
	GG	42	51	**0.53 (0.31–0.92)**^**a**^	**0.022**^**a**^
	GA + GG	146	181	**0.54 (0.36–0.83)**^**a**^	**0.004**^**a**^

^a^Boldface indicates statistical significance.

### Analysis of linkage disequilibrium (LD) and haplotype

Linkage disequilibrium analysis was examined. The analyses showed that LD was moderate between rs1143633 and rs3136558 (D′ value = 0.5814) and between rs3136558 and rs1143630 (D′ value = 0.462), and very low between rs1143633 and rs1143630 (D′ value = 0.0701) in present population (Table 5). Haplotype association analysis of *IL1B* htSNPs with lung cancer risk showed that haplotype4 (rs1143633^A^-rs3136558^C^-rs1143630^A^) was associated with increased risk of lung cancer after adjustment for smoking duration [adjusted-OR (95% CI) = 1.55 (1.07–2.24), *P* = 0.021] and that haplotype2 was marginally associated with increased risk of lung cancer (Table 6).

**Table 5:** Pair-wise linkage disequilibrium analysis for *IL1B* three htSNPs.

Statistic/rs number			
D′ statistic	rs1143633	rs3136558	rs1143630
rs1143633	–	0.5814	0.0701
rs3136558	–	–	0.462
rs1143630	–	–	–

r statistic	rs1143633	rs3136558	rs1143630
rs1143633	–	− 0.4095	0.0365
rs3136558	–	–	0.2568
rs1143630	–	–	–

*P* value	rs1143633	rs3136558	rs1143630
rs1143633	–	0	0.0742
rs3136558	–	–	0
rs1143630	–	–	–

**Table 6:** Haplotype association of *IL1B* htSNPs with lung cancer risk.

Haplotype^a,b^	Construction^c^	Control frequency	Case frequency	OR (95% CI)	*P* value
1	GTC	0.3274	0.295	1.00	–
2	ACC	0.2323	0.2531	1.26 (1.00–1.59)	0.054
3	ATC	0.2328	0.24	1.17 (0.91–1.49)	0.22
4	ACA	0.0693	0.0947	**1.55 (1.07–2.24)**^d^	**0.021**^d^
5	GTA	0.0523	0.04	0.86 (0.51–1.46)	0.58
6	GCC	0.0432	0.0374	0.93 (0.55–1.59)	0.8
7	GCA	0.0353	0.0284	0.91 (0.49–1.69)	0.77
Rare	-	-	-	1.67 (0.49–5.71)	0.42

^a^Adjusted for smoking duration, Global haplotype association *P*-value = 0.1

^b^Three-locus order: rs1143633- rs3136558- rs1143630.

^c^Underlined indicates minor allele.

^d^Boldface indicates statistical significance.

### Analysis of multifactor dimensionality reduction (MDR) approach

Table 7 summarizes the best candidate models of interactions of selected attributes related to lung cancer risk using MDR approach. Three best models were set for whole study group: in the combined interaction analysis of *IL1B, PPP1R13L, POLR1G* and smoking-duration, both the two-factor model and the three-factor model were statistically significant, but the three-factor model (*IL1B* rs3136558, *POLR1G* rs967591 and smoking duration) had a relatively higher values of balanced accuracy overall of 0.6062 and cross-validation consistency of 8/10 that was significant at the *P*-value 0.0100–0.0110. In the conjoined interaction analysis of *IL1B* and smoking-duration, two-factor model, the three-factor model and the fourth-factor model were all statistically significant, but the fourth-factor model (*IL1B* rs1143633, rs3136558 and rs1143630 and smoking duration) had a relatively higher values of balanced accuracy overall of 0.6112 and cross-validation consistency of 10/10 that was significant at the *P*-value 0.0040–0.0050. In the joint interaction analysis of *IL1B, PPP1R13L, POLR1G*, both the two-factor model and the three-factor model were statistically significant, but the three-factor model (*IL1B* rs1143633, *PPP1R13L* rs1970764, *POLR1G* rs735482) had a relatively higher values of balanced accuracy overall of 0.5973 and cross-validation consistency of 10/10 that was significant at the *P*-value 0.0130–0.0140. Smoking-duration presented interaction main-effect in model consisting of *IL1B* htSNPs-*PPP1R13L* and *POLR1G* SNPs-smoking duration or *IL1B* htSNPs-smoking duration. *IL1B* rs1143633 presented interaction main-effect in model consisting of *IL1B* htSNPs. For histopathology study subgroup: only conjoined interaction analysis of *IL1B* and smoking-duration was performed. In the subgroup of squamous cell carcinoma, both the two-factor model (*IL1B* rs1143633 and smoking duration) and the four-factor model (*IL1B* rs1143633, rs3136558 and rs1143630 and smoking duration) had relatively higher values of balanced accuracy overall of and cross-validation consistency of 10/10 that were statistically significant at the *P*-values, and smoking duration showed obvious main effects. In the subgroup of other histopathology, the two-factor model had statistical significance. In the subgroup of lung adenocarcinoma, no interaction was identified.

**Table 7:** The best candidate models of interactions of selected attributes related to lung cancer by MDR approach.

Model factor^a,b^	Attribute	Bal. Acc.^c^ overall	Bal. Acc. CV^d^ training	Bal. Acc. CV testing	CV consistency	*P* value^e^
Whole group						
Lung cancer						
*IL1B* + *PPP1R13L* + *POLR1G* + smoking-duration						
One-factor	Smoking	0.5708	0.5708	0.5708	10/10	**0.0040–0.0050**
Two-factor	Smoking					
	rs1143633	0.5849	0.5866	0.5649	5/10	**0.0090–0.0100**
Three-factor	Smoking					
	rs3136558					
	rs967591	0.6062	0.6084	0.5602	8/10	**0.0100–0.0110**
*IL1B* + smoking-duration						
One-factor	Smoking	0.5708	0.5708	0.5708	10/10	**< 0.0010**
Two-factor	Smoking					
	rs1143633	0.5849	0.5862	0.5721	6/10	**< 0.0010**
Three-factor	Smoking					
	rs1143633					
	rs1143630	0.5944	0.5964	0.5619	8/10	**< 0.0010**
Fourth-factor	Smoking					
	rs1143633					
	rs3136558					
	rs1143630	0.6112	0.6139	0.5588	10/10	**0.0040–0.0050**
*IL1B* + *PPP1R13L* + *POLR1G*						
Two-factor	rs1143633					
	rs1970764	0.5682	0.5689	0.5507	9/10	**0.0440–0.0450**
Three-factor	rs1143633					
	rs1970764					
	rs735482	0.5973	0.5987	0.5576	10/10	**0.0130–0.0140**
*IL1B*						
One-factor	rs1143633	0.5451	0.5451	0.5451	10/10	**0.0290–0.0300**
Histopahtology subgroup						
Lung squamous cell carcinoma						
*IL1B* + smoking-duration						
One-factor	Smoking	0.6315	0.6318	0.6227	10/10	**< 0.0010**
Two-factor	Smoking					
	rs1143633	0.6532	0.6533	0.6472	10/10	**< 0.0010**
Three-factor	Smoking					
	rs1143633					
	rs1143630	0.6619	0.6645	0.6123	5/10	**< 0.0010**
Fourth-factor	Smoking					
	rs1143633					
	rs3136558					
	rs1143630	0.6785	0.6827	0.5871	10/10	**0.0030–0.0040**
Other						
*IL1B* + smoking-duration						
Two-factor	Smoking					
	rs3136558	0.6178	0.6181	0.6073	10/10	**0.0090–0.0100**

^a^Analyzed by MDR 3.0.3. dev. Jar, data of *PPP1R13L* + *CD3EAP* from previous study (Yin et al.^18^).

^b^Only list statistical significant models.

^c^Balanced accuracy.

^d^Cross-validation.

^e^*P* value based on 1000 permutation test. Boldface indicates statistical significance.

**Figure 1:**
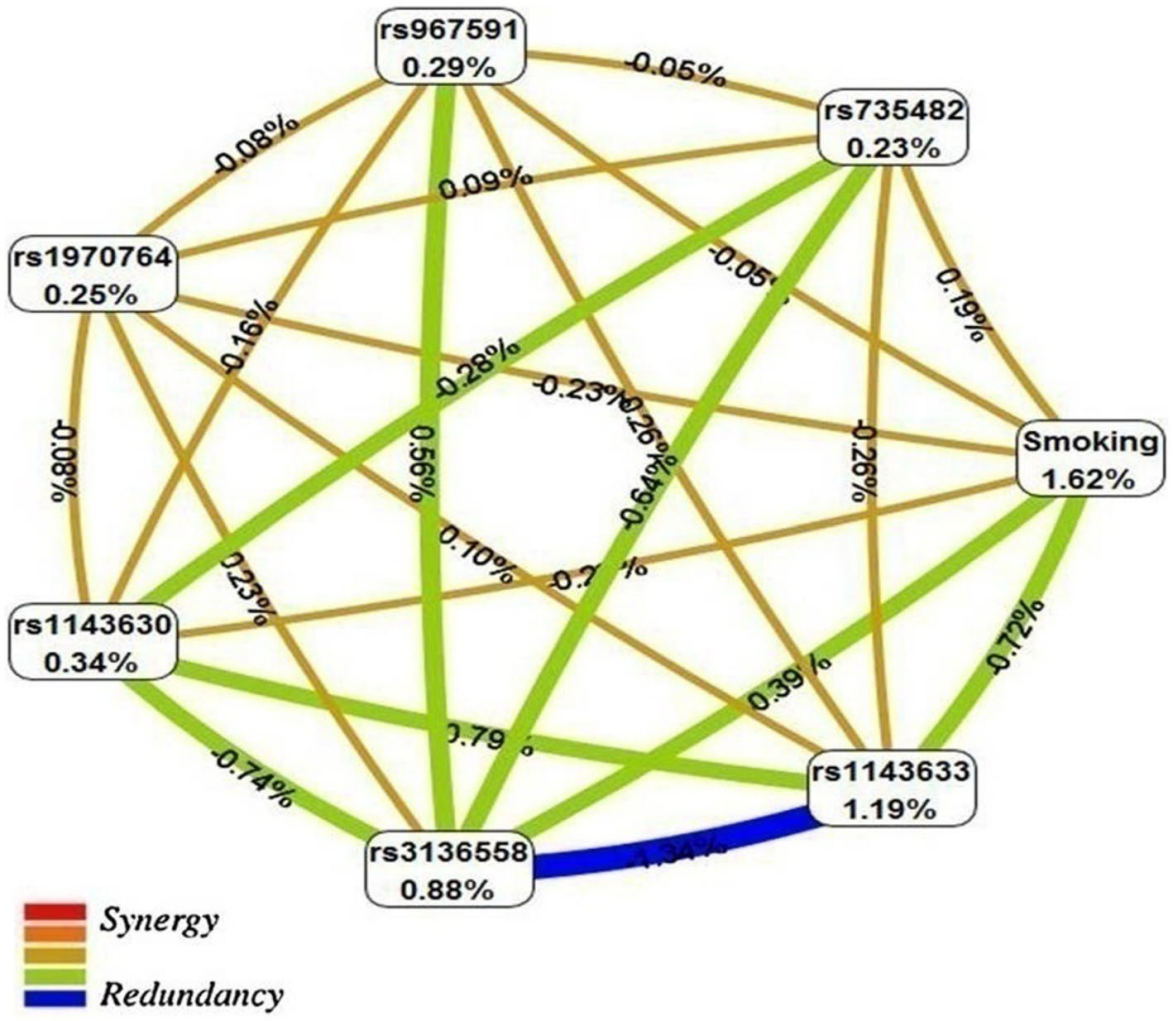
Interaction entropy model. This graphical model, describes the percent entropy that is explained by each selected attributes or pair-wise combination in our study population. Positive percent entropy indicates information gain (IG) or synergy and negative percent indicates lack of information gain (IG) or redundancy. Schematic coloration used in the visualization tools represents a continuum from synergy (i.e. non-additive) to redundancy. Red represents a high degree of synergy interaction, orange a lesser degree (both colors are not apparent in the current analysis), brown represents medium-level interaction or independence; and green and blue represent redundancy between the markers. This image was created by MDR software (3.0.3. dev. Jar) (https://sourceforge.net/projects/mdr/)^36^.

Figure 1 shows the interaction entropy model from the interaction analysis of *IL1B* htSNPs, *PPP1R13L* and *POLR1G* SNPs and smoking-duration built using the MDR software. The entropy-based model indicated that some values between 7 attribute interaction presented medium-level interaction or independence whereas degree of synergy interaction was not apparent in the current analysis between 7 attributes.

## Discussion

### Studies addressing *IL1B* SNPs in cancer

Previous epidemiology studies have identified association of *IL1B* SNPs with cancers risk but with inconsistent results^6–13^. A Caucasian-Danish prospective study showed that variant allele carriers of *IL1B* SNP rs1143627 (− 31T>C) were at increased risk of lung cancer [Dominant model: IRR (incidence rate ratio) (95% CI) = 1.51 (1.08–2.12)]^7^. An Asian-Chinese case–control study reported *IL1B* SNPs rs16944 (− 511G>A) and rs1143623 (C>G) was associated with decreased breast cancer risk [co-dominant model: OR (95% CI) = 0.60 (0.41–0.90), *P* = 0.034; co-dominant model: OR (95% CI) = 0.65 (0.45–0.94), *P* = 0.023]^6^. A Russian case–control study revealed a significant association between *IL1B* SNP rs1143623 (− 1473G>C) and risk of rectal cancer [co-dominant model: OR (95% CI) = 1.67 (1.06–2.63), *P* = 0.048]^8^. An Asian-Chinese case–control study reported association between *IL1B* SNP rs1143634 (+ 3954C>T) and gastric cancer risk [co-dominant model: OR [95% CI) = 6.93 (3.13–15.36)]^4^. A Asian-Korean study suggested that *IL1B* SNPs rs1143633 (A>G) and rs1143627 (T>C) protected against hepatocellular carcinoma [dominant model: OR (95% CI) = 0.59 (0.37–0.94), *P* = 0.027; dominant model: OR (95% CI) = 0.56 (0.34–0.91), *P* = 0.019] and *IL1B* SNP rs3917356 (G>A) increased the risk of hepatocellular carcinoma [recessive model: OR (95% CI) = 2.58 (1.53–4.33), *P* < 0.001]^9^. A Caucasian-Danish prospective case-cohort study showed that variant genotypes of *IL1B* SNPs rs4848306 (− 3737C>T) and rs1143623 (− 1464G>C) were associated with colorectal cancer risk [dominant model: IRR (95% CI) = 0.81 (0.68–0.97), *P* = 0.02; dominant model: IRR (95% CI) = 1.22 (1.04–1.44), *P* = 0.02]^10^.

A Caucasian-American community-based case–control study (The Prostate, Lung, Colorectal, and Ovarian (PLCO) Cancer Screening) reported the common rs16944 and rs1143634 SNPs of *IL1B* did not seem to play a role in prostate cancer risk^11^. A Chinese case–control study reported that *IL1B* SNP rs1143634 was not associated with gastric cancer risk^12^. A Asian-Chinese case–control study of inflammation-related genes involved in wound healing reported that studied *IL1B* SNPs were not associated with oesophageal squamous cell carcinoma^13^.

### Main findings, implications and strengths of study

In this Northeastern-Chinese case–control study, we examined three htSNPs tagging 95% of the haplotyping diversity of *IL1B* known to be involved in the inflammatory response and associated with cancer risks in previously studies. To the best of our knowledge, this is the first study to evaluate three htSNPs tagging 95% of the haplotyping diversity of *IL1B* and to assess specific interactions between *IL1B* htSNPs, *PPP1R13L*, *POLR1G* risk SNPs and smoking-duration in relation to a lung cancer risk.

From a classical case–control approach in whole study, our main finding is that variant G-allele of *IL1B* SNP rs1143633 (A>G) associated with lower risk of lung cancer under dominant model and that variant C-allele of *IL1B* SNP rs3136558 (T>C) was at increased risk of lung cancer. When stratified by smoking-duration, *IL1B* SNP rs1143633 was specifically associated with lung cancer risk among long-term smokers (> 20 years). When stratified by histopathology, it should be noted that the studied *IL1B* htSNPs were only associated with risks among patients with lung squamous cell carcinoma and not among patients with lung adenocarcinoma. These results again suggest that the pathogenesis of the two subtypes may be different in genetic factors and gene changes^21^. The haplotype analysis of *IL1B* three htSNPs revealed positive association with lung cancer risk for the haplotype4 encompassing the variant alleles of rs3136558 and rs1143630. Haplotype2, which also encompasses the wild-type allele of rs1143633 and the variant allele of rs3136558, was also marginally associated with increased lung cancer risk. The other haplotypes encompassing the variant allele of rs1143630 were not associated with lung cancer risk. This suggests that haplotypes2 and 4 are in linkage with the functional *IL1B* polymorphism. The present result for *IL1B* SNP rs1143633 replicates for the finding from hepatocellular carcinoma study in Asians-Korean^9^. The present result of *IL1B* SNP rs3136558 (T>C) agrees with the finding that variant-allele carriers of *IL1B* SNP were at increased lung cancer risk among Caucasus-Danes^7^.

We did not correct for multiple testing. In this study, we included 3 SNPs, and thus, it could be argued that the threshold for statistical significance should be 0.05/3 = 0.0167. However, the current study is hypothesis driven, and the SNPs were selected to be in linkage disequilibrium with 95% of the genetic variation in *IL1B*.

The integration of genetic variants in risk prediction models beyond the traditional epidemiological covariates has been considered as the way forward in lung cancer risk prediction modeling^22^. Using a MDR approach: for whole study group, smoking-duration or *IL1B* rs1143633 was observed respectively as single main effect in one-factor model. The values of balanced accuracy overall and cross-validation consistency raised along with the increasing number of the factors. This phenomenon indicates the presence of interaction, meaning that the effect change of smoking-duration or rs1143633 at different levels depends on the level of another or several factors. Its existence shows that the effects of several factors studied simultaneously are not independent of each other. Special interactions between smoking duration, *IL1B* rs3136558 and *POLR1G* rs967591; smoking-duration, *IL1B* rs1143633, rs3136558 and rs1143630; and *IL1B* rs1143633, *PPP1R13L* rs1970764 and *POLR1G* rs735482 were observed in relation to lung cancer risk. The medium-level interactions were found between most markers. For histopathology study subgroup: smoking-duration was as main effect and positive interactions were only seen in subgroup of lung squamous cell carcinoma. These results add new evidence to our previous study^17^. The results again suggests that lung squmacarcinoma cell carcinoma displays the strongest relation with tobacco-smoking than lung adenocarcinoma^17^. Overall MDR results show that smoking duration as the main effect and the interactions between *IL1B* htSNP and *PPP1R13L* SNP and *POLR1G* SNP and smoking duration play critical roles in the occurrence of lung cancer and lung squamous cell carcinoma.

There is evidence of causal relationships between chronic infection, inflammation, and cancer^23^. An inflammatory microenvironment is an essential component of the tumor microenvironment (TME)^20^. The lung presents a unique milieu in which tumors progress in collusion with the TME. Inflammation plays an important role in the pathogenesis of lung cancer, and pulmonary disorders in lung cancer patients such as chronic obstructive pulmonary disease (COPD) and emphysema, constitute co-morbid conditions and are independent risk factors for lung cancer^24^. Chronic inflammation is a key feature of COPD and could be a potential driver of lung cancer development^25^. The chronic inflammatory microenvironment is associated with the release of various pro-inflammatory and oncogenic mediators including cytokines IL1B^20^. Excessive and uncontrolled releases of pro-inflammatory cytokines such as IL1B were increased in severe corona-virus disease 2019 (COVID-19) patients^26^. Environmental and occupational toxicants may induce pulmonary inflammation^27–29^. Chronic inflammation has been linked to several human diseases and also to initiation and promotion of cancer. High-expression of the promoter of *IL1B* SNP rs1143627 (− 31T>C) was induced in the human lung epithelial NCI-H2009 cells (Human lung adenocarcinoma cell line) treated with cigarette-smoke condensate^30^. Release of inflammasome products, such as IL1B and cytokine storms are hallmarks of COVID-19 infection and smoking may critically exacerbate COVID-19-related inflammation^31^. Present interaction study have added evidence that related to inflammation and immunity *IL1B*, which is independent or combined with other factors such as smoking, is involved in lung cancer risk.

### Potential functional roles of selected *IL1B* htSNPs

SNP Function Prediction (FuncPred)^32^ indicated that *IL1B* rs1143630 has significant conservation score = 0.004 in three htSNP analyses about nsSNP (non-synonymous coding SNPs), splicing regulation, stop Codon, polyphen prediction, SNPs3D prediction, TFBS (transcription factor-binding site) prediction, miRNA binding site prediction, regulatory potential score, and conservation score by present data of SNPinfo Web Server. However we observed rs1143633 was the most important htSNP in this study, and we identified haplotype4 as a candidate to be in linkage with the functional SNP. Several functional SNPs have been identified in *IL1B*^14^.

### Limitations

We have several study limitations. Power-test analyses for current study showed that for rs1143633, we had 91% chance of detecting OR = 0.67 at the 0.05 significant level using two-sided tests under the dominant model, showing that the sample size is reasonable and can meet the reasonable confidence level conditions. We had 80% or 81% chance for rs3136558 or rs1143630 respectively, detecting OR = 1.4 at the 0.05 significant level using two-sided tests under the dominant model, indicating that further larger population-based studies are warranted to confirm present findings. In addition, the haplotype analysis suggests that the studied SNPs are in linkage with the functional polymorphism. Thus functional studies of the polymorphisms under study would reveal whether the polymorphisms are functional or whether the observed associations are due to linkage with the functional polymorphism. Although the current research improves the efficiency of controlling confounding factors by matching age, sex and ethnic between cases and controls, which cannot directly control other confounding factors such as smoking-duration.

## Conclusions

Our findings suggest that *IL1B* SNP rs1143633 may associate with lower risk of lung cancer, confirming previously identified marker; *IL1B* SNP rs3136558 and haplotype4 consisting *IL1B* htSNPs (rs1143633^A^-rs3136558^C^-rs1143630^A^) may associate with increased risk of lung cancer; interactions of *IL1B* with *POLR1G* or *PPP1R13L* or smoking-duration, which is independent or combined, may involve in risk of lung cancer and lung squamous cell carcinoma. These interesting findings should be sought in further validation with larger prospective cohorts. These could be used as a clinical biomarkers in lung cancer.

## Materials and methods

### Study population

1260 individuals were enrolled in this hospital-based case–control study including 627 cases and 633 controls as previously report^18^. The patients with lung cancer were diagnosed based on standard clinical and histological criteria. Eligible cases were previously untreated (no chemotherapy or radiotherapy for cancer prior to recruitment). Cancer-free controls (matched on: sex same, age ± 3 years and ethnic same) were selected from the orthopedics wards in the same area. Demographic and covariate data were obtained from medical records and questionnaires by personal interview with professional physicians. All participants were unrelated ethnic Han Chinese from Northeast China. Stratification criteria were defined as follows: age (10 years intervals), sex, family history of cancer, smoking duration (20 years intervals) and histopathology (3 subgroups).

### htSNPs in *IL1B* gene determined

htSNPs of the *IL1B* gene were achieved from region of chromosome 12 of the International HapMap Project [http://www.hapmap.org, HapMap Data Rel 27 PhaseII + III, Feb09, on NCBI B36 assembly, dbSNP [the dbSNP database (https://www.ncbi.nlm.nih.gov/snp/) b26], by applying the TagSNPs software online and approaches of the algorithm-Tagger-pairwiseTagging. Qualified criteria: r2-cut off at 0.8 and minor allele frequency (MAF)-cut off at 0.05 in Han Chinese in Beijing (HCB) samples. Three htSNPs (rs1143633, rs3136558 and rs1143630) (the dbSNP database: https://www.ncbi.nlm.nih.gov/snp/?term=rs1143633, https://www.ncbi.nlm.nih.gov/snp/?term=rs3136558 and https://www.ncbi.nlm.nih.gov/snp/?term=rs1143630) were selected representing 95% of the common haplotype diversity across the *IL1B* gene. Table 2 displays the information of *IL1B* three htSNPs and three risk SNPs in Chr19q13.3 sub-region. Three risk SNPs of Chr19q13.3 were previously reported^16,18,33^. The data of three risk SNPs in *PPP1R13L* and *POLR1G* were used for analyses of gene–gene and gene–gene–environment interaction in current study.

### DNA isolation and genotyping

A volume of 5 mL of whole blood with ethylenediamine tetraacetic acid (EDTA) anticoagulation was taken from each volunteer. Genomic DNA of whole blood samples was drawn with the Puregene DNA Isolation Kit or FlexiGene DNA kit 250 (Gentra Systems, Minneapolis, MN, USA or Qiagen, Germany) following the product' s instructions. Genotyping of rs1143633 (A>G), rs3136558 (T>C) and rs1143630 (C>A) of the *IL1B* gene was executed with the genotyping assay of ligase detection reaction coupled with polymerase chain reaction (LDR-PCR) as previously published^34^ in Shanghai Generay Biotechnology Co. Ltd. (P. R. China). Genotypes of *PPP1R13L* rs1970764 (A>G) and *POLR1G* rs967591 (G>A) and rs735482 (A>C) have been previously reported^16^. The software of Primer Premier 5.0 was used for design primers. The sequences (5′–3′) of primers and probes of *IL1B* three htSNPs are displayed in Table 8. Each group of LDR probes consisted of 1 common probe and 2 discriminating probes for the 2 alleles. For the PCR reactions, the DNA concentration was 50 ng–100 ng/μL and DNA purity was OD260/OD280 = 1.8–2.0. The genotyping procedure was in summary: performed PCR reactions, completed LDR reactions and sequenced LDR products. The genotyping call-rate was 96.35% for the *IL1B* three htSNPs. As quality control: pure water was used as negative control and 20% samples including cases and controls were genotyped twice, yielding 100% identical results.

**Table 8:** The sequences (5′–3′) of primers and probes for *IL1B* three htSNPs examined.

rs Number	Primers	Probes
rs1143633		
Forward primer	CTACTGGTGTTGTCATCAGAC	
Reverse primer	AGCTTTTTTGCTGTGAGTCCC	
Common probe		-P-CCTCGCCTCACGAGGCCTGCCCTTC-HEX-
Discriminating probe G		TTTTCCTCCAAGAAATCAAATTTTGCCG
Discriminating probe A		TCCTCCAAGAAATCAAATTTTGCCA
rs3136558		
Forward primer	TCGCCATGTTGGCCAGGCTGG	
Reverse primer	GTGGTCCTGCCAGGAACCATG	
Common probe		-P-CTTTAGAAGCTCGGGATTCTTTCAATTT-HEX-
Discriminating probe C		TTTTCACGCCTGGCCCAGAGAGGGATGAC
Discriminating probe T		TTTTTTTCACGCCTGGCCCAGAGAGGGATGAT
rs1143630		
Forward primer	GAATAGCCTGTAAGGTGTCAG	
Reverse primer	GGATGCAGTAAGCCAAGATTG	
Common probe		-P-CTTAACCTCCTTGAGCTTCAGAGAGTTTTTTTTT-FAM-
Discriminating probe A		TTTTTTTTTTTTCCTGCTGTGTGCCCTTGAGTACACA
Discriminating probe C		TTTTTTTTTTTTTTTCCTGCTGTGTGCCCTTGAGTACACC

### Statistical analysis

Selected characteristics of cases and controls, allele frequencies, genotype frequencies, Hardy–Weinberg equilibrium, co-dominant model; dominant model; recessive model; over-dominant model; and log-additive model for case–control association of each single-locus, haplotype associations, and pair-wise LD, unconditional logistic regression for measurement of OR (95% CI) after adjusting smoking-duration, The Shapiro–Wilk test and Mann–Whitney U test were explored employing SPSS© v16.0 (SPSS Inc, Chicago, IL, USA) or SNPStats program^35^. Akaike’s Information Criterion (AIC) is a standard to measure the goodness of fit for statistical model. AIC criterion was used: give priority to model with the lowest AIC value^35^. Haplotypes with frequency < 0.01 among both cases and controls were excluded from the analysis. The interaction analyses of gene–gene and gene–gene-smoking duration in relation to lung cancer risk were conducted employing platform of MDR. This software (3.0.3. dev. Jar)^36^ is an updated version where permutation testing has been added into the main MDR program. The MDR method is nonparametric and free model. MDR has rational power for identifying interactions between two or more loci in relatively small samples. MDR has excellent power for identifying high-order gene–gene interactions. MDR can be directly used to case–control and discordant-sib-pair studies^36^. MDR conducts selection and evaluation of model by cross-validation and permutation-test. Balanced accuracy cross-validation training and balanced accuracy cross-validation testing indicate the accuracy rate in the training set and the testing set, respectively. Whose range is 0–1. The larger the number, the higher the accuracy rate. Cross-validation consistency indicates the consistency rate of cross-validation. Permutation-test = 1000 was set according to the instructions^36^. The *P* value was less than 0.05 was considered statistically significant. The possible functionality of *IL1B* three htSNPs was assessed using the web tool: SNPinfo^31^ in silico analysis. Power test was examined employing online statistical software: Unmatched Case/Control Studies (https://www.stat.ubc.ca/~rollin/stats/ssize/caco.html).

### Ethics approval

The study protocol was approved by Human Genetic Resource Administration of China (HGRAC) (no. [2001] 015) and was conducted in accordance with the Helsinki Declaration. Every study participant agreed to participate in the study.

## Data Availability

The information of selected htSNPs across the *IL1B* gene was from the dbSNP database: https://www.ncbi.nlm.nih.gov/snp/?term=rs1143633, rs3136558 and rs1143630. All data generated during this study are included in this published article. The data that support the findings of this study are available from the corresponding author upon reasonable request.
